# Potentiation of anti-glioma immunity induced by oncolytic adenovirus Delta-24-RGD through viral expression of immune co-stimulator OX40 ligand

**DOI:** 10.1186/2051-1426-3-S2-P337

**Published:** 2015-11-04

**Authors:** Hong Jiang, Karen Dwyer, Laura Bover, Frederick Lang, Candelaria Gomez-Manzano, Juan Fueyo

**Affiliations:** 1 MD Anderson Cancer Center, Houston, TX, USA; 2 UT MD Anderson Cancer Center, Houston, TX, USA

## Background

Oncolytic viruses are promising alternative cancer therapies that can cause anti-cancer immunity. We found Delta-24-RGD, an oncolytic adenovirus currently undergoing Phase I clinical trial in patients with recurrent glioblastomas, induced anti-glioma immunity in an immunpcompetent mouse model. We hypothesized that the anti-cancer immunity mediated by the virus could be augmented through expressing the immune co-stimulator OX40 ligand (OX40L) by the virus to enhance the antigen-presenting function of the cancer cells.

## Methods

Thus we constructed Delta-24-RGDOX which included an OX40L-expressing cassette in the backbone of Delta-24-RGD. The new virus expressed OX40L efficiently in cultured cancer cells and in implanted glioma cells in mice while kept the same replication efficiency as Delta-24-RGD.

## Results

Like its precedessor Delta-24-RGD, Delta-24-RGDOX induced immunogenic cell death in glioma cells. Importantly, compared to Delta-24-RGD, Delta-24-RGDOX induced enhanced anti-glioma activity in immunocompetent glioma models (means: 28.5 versus 17 days, *P* < .0001) but not in an immunodeficient model. Since glioma cells expressed high levels of PD-L1, we combined Delta-24-RGDOX with anti-PD-L1 antibody. Impressively, it resulted in 100% long-term survival in the treated mice while the virus alone only induced 25% long-term survival (*P* = 0.001). Further studies revealed that Delta-24-RGDOX induced higher levels of lymphocyte infiltration at the tumor sits (*P* < .001), greater anti-tumor activity of the lymphocytes (*P* < .05) and proliferation of tumor-associated antigen specific lymphocytes (*P* = .0002) than Delta-24-RGD.

## Conclusions

Collectively, our data demonstrate that oncolytic viruses carrying immune co-stimulatory ligands and its combination with antibodies mainly targeted to cancer cells may constitute powerful and safer alternatives to the antibodies and other strategies targeting immune checkpoints in cancer therapy and warrant the development of clinical trials in cancer patients in the near future.

